# Advancing Lipid Management in Europe: Insights from the WHF Regional Roundtable Series and Country Case Studies

**DOI:** 10.5334/gh.1548

**Published:** 2026-04-16

**Authors:** Lana Raspail, Sean Taylor, Jeanine Roeters Van Lennep, José R. González-Juanatey, Kausik K. Ray, Raul D. Santos

**Affiliations:** 1 World Heart Federation, Switzerland; 2 Department of Internal Medicine, Erasmus MC Cardiovascular Institute, University Medical Center Rotterdam, Rotterdam, The Netherlands; 3 Centro de Investigación Biomédica en Red de Enfermedades Cardiovasculares (CIBERCV), Madrid, and University of Santiago de Compostela, Santiago de Compostela, Spain; 4 Imperial Centre for Cardiovascular Disease Prevention, Department of Primary Care and Public Health, Imperial College London, School of Public Health Building, United Kingdom; 5 Imperial Clinical Trial Unit (ICTU)-Global, Imperial College London, United Kingdom; 6 Hospital Israelita Albert Einstein and Heart Institute (InCor) University of Sao Paulo Medical School Hospital, São Paulo, Brazil

**Keywords:** cardiovascular disease, lipids, health policy, screening

## Abstract

Elevated cholesterol remains a major modifiable risk factor for atherosclerotic cardiovascular disease (ASCVD), yet lipid targets are unmet in many high- and very-high-risk individuals across Europe. This gap persists despite strong evidence, established guidelines, and effective therapies. To address this, the World Heart Federation (WHF) convened a Regional Europe Roundtable Series, bringing together clinicians, policymakers, researchers, and patient representatives. This review synthesises insights from meetings held in July and August 2025, alongside country case studies.

Key barriers include low awareness, statin misinformation, fragmented care pathways, limited data systems, inequitable access to therapies, and inconsistent policies for familial hypercholesterolaemia detection and management. Despite these challenges, scalable solutions exist, including national strategies, screening programmes, digital referral systems, and integrated prevention initiatives. Strengthening policies, data systems, primary care capacity, public education, and equitable access to therapies will be essential to close the implementation gap in lipid management across Europe.

Summary of challenges and solutions for lipid management in Europe, including unmet targets, fragmented care, limited data, and inequitable access, alongside proposed actions such as national strategies, screening programmes, digital tools, and improved therapy access.

**Graphical Abstract:**
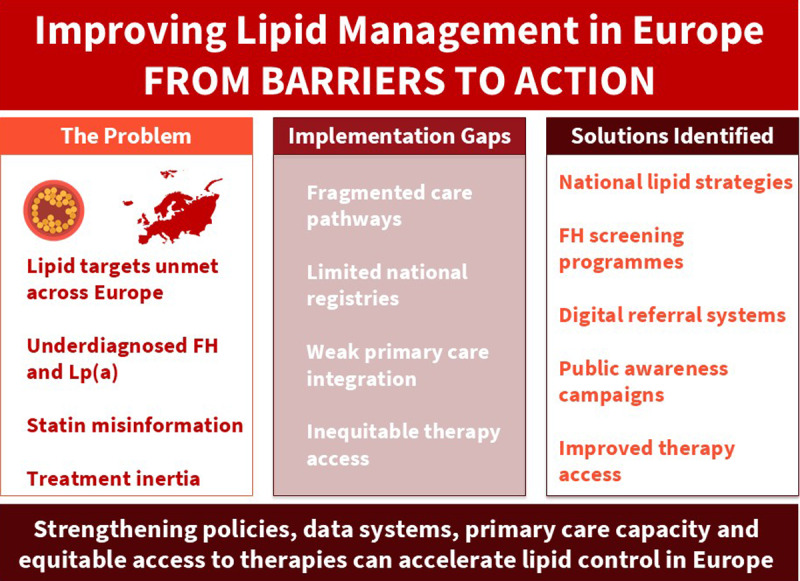
Key challenges and solutions for lipid management in Europe.

## Introduction

Elevated cholesterol remains one of the most important modifiable risk factors for atherosclerotic cardiovascular disease (ASCVD), yet lipid targets are still not met for a large proportion of high- and very-high-risk individuals across Europe and the world. This gap persists despite strong evidence, clear guidelines, and the availability of inexpensive and effective treatments. Multiple policy, health system, cultural, and educational barriers impede successful implementation (1).

To accelerate progress, the World Heart Federation (WHF) launched a Regional Europe Roundtable Series aimed at bringing together key stakeholders to identify barriers, share best practices, and develop actionable, country-level and regional recommendations for improving lipid management. This review synthesises insights from two meetings:

1. A virtual roundtable held on 8 July 2025.
2. An in-person roundtable held in Madrid, Spain, on 30 August 2025 during the European Society of Cardiology (ESC) Congress.

The purpose of this review is to consolidate the discussions, highlight cross-cutting themes, and propose practical, regionally relevant strategies for European countries seeking to strengthen the detection, treatment, and control of dyslipidaemia.

## Overview of the WHF Roundtable Series

The WHF Regional Roundtable Series seeks to enhance the implementation of guideline-based lipid management by convening national and regional experts to discuss barriers, best practices, and opportunities for collaboration. The series contributes to WHF’s broader mission to support countries in adapting the WHF Roadmap on Cholesterol and translating evidence into effective system-level action. The sessions brought together cardiologists, lipidologists, patient organisations, government representatives, and industry observers. The meetings prioritised shared learning and pragmatic solutions that could be adapted to diverse European contexts.

## Key Themes from the Roundtable Discussions

### 1. Persistent gaps in detection and management of inherited lipid disorders

Both meetings identified familial hypercholesterolaemia (FH) and lipoprotein(a) (Lp(a)) as major yet under-recognised contributors to cardiovascular risk across Europe. Recent estimates suggest that FH affects roughly 1 in 250–300 individuals globally, meaning several million people in Europe alone (2).

#### Underdiagnosis and undermanagement of FH

Despite this high prevalence and the availability of effective therapies, FH remains underdiagnosed and undertreated in most European countries. Participants reported significant gaps in FH detection despite guideline recommendations. Barriers include limited primary care awareness, fragmented referral pathways, absent national registries, and insufficient funding for genetic testing. Several countries still lack systematic cascade screening programmes.

#### Lp(a): A neglected risk factor

Lp(a) has been established as a causal, independent risk factor for ASCVD and aortic valve stenosis, with approximately 20–25% of the population having elevated levels. The 2022 European Atherosclerosis Society consensus statement recommends that Lp(a) be measured at least once in adulthood as part of comprehensive risk assessment, especially in those with premature ASCVD or a strong family history (3). Experts highlighted the issue of inadequate awareness and limited routine testing of Lp(a), despite its strong association with premature ASCVD. Most countries do not routinely include Lp(a) measurement in standard cardiovascular risk assessment, even when family history or unexplained events warrant testing. Participants agreed that integrating Lp(a) measurement into national cardiovascular disease (CVD) prevention programmes and pathways is an opportunity for improvement.

### 2. Statin underuse, misinformation, and the nocebo effect

A major cross-country challenge is the underuse of statins despite their affordability and effectiveness. Meta-analyses of randomised statin trials show robust reductions in major vascular events and mortality across a wide range of baseline risk and low-density lipoprotein cholesterol (LDL-C) levels (4). Roundtable participants noted persistent misconceptions about statin safety among the public, and in some cases, among healthcare providers, often fuelled by sensational media reporting. Statin ‘intolerance’ driven by the nocebo effect, together with therapeutic inertia and inconsistent guideline implementation in primary care, contribute to a large pool of undertreated high-risk patients across Europe. These issues were reported by participants from Germany, the Netherlands, and other countries facing cultural resistance or misinformation campaigns.

### 3. Inequities in access to advanced therapies

Advanced therapies such as PCSK9 monoclonal antibodies and small interfering RNA (siRNA) can reduce LDL-C by 50–60% on top of statins and ezetimibe (5). Access to these therapies varies widely across Europe. Some countries, such as Spain, Poland and Cyprus, have secured reimbursement for selected high-risk individuals, while others lack coverage or have withdrawn reimbursement, limiting access even for patients with severe FH or uncontrolled LDL-C on maximally tolerated statin therapy. Participants agreed that these access challenges reflect broader issues of inconsistent health system financing, regulatory delays, and limited use of cost-effectiveness data in national decision-making processes.

### 4. Systemic barriers: Fragmented care and weak primary care integration

Many countries face structural challenges that weaken lipid management across the continuum of care. These include fragmented care pathways hindering referrals and follow-up, limited integration of electronic systems between primary and secondary care, and scarce national data on screening, diagnosis, treatment, and control rates (6). Inadequate workforce training in lipid management, especially in primary care, further undermines guideline adoption.

Poland, Kazakhstan, Latvia, and Cyprus highlighted obstacles related to workforce capacity, inconsistent quality metrics, and poor interoperability of systems. These local insights align with the broader implementation gaps described in the WHF Cholesterol Roadmap and contemporary implementation science literature, underscoring the need for structured quality improvement and health system strengthening approaches. A summary of the key barriers identified during the roundtable series is in Table 1.

**Table 1:** Key barriers to effective lipid management in Europe.


BARRIER CATEGORY	DESCRIPTION	EXAMPLES FROM ROUNDTABLES

**Awareness and Beliefs**	Low public and provider awareness; cholesterol scepticism; statin misinformation	Cholesterol scepticism in Germany and Netherlands; nocebo effects; Lp(a) not commonly measured

**Systemic/Structural**	Fragmented care pathways; weak primary care integration; lack of national registries	Kazakhstan, Latvia: Limited integration; many countries lacking FH/Lp(a) registries

**Access and Reimbursement**	Inequitable access to novel treatments like PCSK9i, combination therapy	Reimbursement withdrawn in Germany; limited access in many countries; Spain, Cyprus and Poland showing strong access

**Policy and Funding**	Inconsistent national commitments; defunding of FH screening	Netherlands: previous FH screening defunded; policymaker resistance to infant screening

**Data Availability**	Sparse national data on screening, diagnosis, treatment, control	Many countries lack disaggregated data; limited cost-effectiveness evidence


## Best Practices and Success Stories

Despite shared challenges, several countries represented at the roundtables presented notable achievements (Table 2) demonstrating scalable models for improvement.

**Table 2:** Best practices and successful models identified across Europe.


COUNTRY	SUCCESSFUL STRATEGY	KEY COMPONENTS

**Spain**	National Cardiovascular strategy	Digital integration; quality benchmarking; universal FH screening; broad reimbursement

**Poland**	National FH programmes	Universal screening ≥ 6 years; network of lipid centres; school-based education

**Norway**	FH advisory and referral optimisation	General Practitioner (GP) outreach; referral flyers; online tools; tripled genetic testing

**Latvia**	Digital self-referral triage	Electronic questionnaire linked to e-ID; guides GP vs specialist referral

**Cyprus**	Personalised communication models	Patient personas; targeted information/screening campaigns; behavioural insights and citizen co-design

**Greece**	National primary prevention programme	Screening of lipid profile (including Lp(a)) in all primary prevention subjects aged 30–70 years

**Czechia**	Universal preventive health check-ups	Comprehensive cardio-reno-metabolic disease screening, including FH and Lp(a) universal testing

**Bulgaria**	Integrated Cardiovascular prevention and digital surveillance strategy	Local adaptation of European Cardiovascular prevention plans; targeted high-risk screening programmes (ProAction BG, REVEALED); EHR-linked lipid and LLT monitoring; national lipid centres; early FH identification; personalised digital public education


### Spain: A comprehensive national cardiovascular strategy

Spain is an exemplar of integrated lipid management, with:

- A national cardiovascular plan including cholesterol management, equity, and quality metrics.
- A benchmarking system that highlights regional differences and drives policy action.
- Interoperable digital systems linking hospitals and primary care.
- Universal FH screening in children and broad reimbursement of lipid-lowering therapies.

### Poland: Nationally funded FH programmes

Poland has:

- Government-funded universal FH screening from age 6.
- A national multidisciplinary lipid programme and expanding network of lipid centres.
- Recent reimbursement of PCSK9 inhibitors supported by national data.
- School-based health education initiatives to improve awareness and long-term adherence.

### Norway: Effective FH referral models

A government-funded FH advisory unit improved detection through:

- GP engagement
- Flyers outlining referral pathways
- Online tools

This approach tripled genetic testing referrals.

### Latvia: Digital self-referral pathways

Latvia developed a digital questionnaire linked to electronic identification (ID), guiding individuals toward GP or specialist consultation—a low-cost, scalable innovation.

### Cyprus, Romania and Bulgaria: Personalised communication models

Research led efforts in Cyprus and Romania in developing personalised awareness and screening campaigns for FH based on behavioural segmentation (‘personas’), co-created with citizens and patients in both countries. Bulgaria launched digital awareness tools with personalised risk feedback.

### Czechia: Universal preventive health check-ups

As a step of implementation of National Cardiovascular Disease Health Plan, the system of preventive health check-ups has been expanded and comprises systematic screening of cardio-reno-metabolic diseases including assessment of Lp(a) in all adults at least once a lifetime. Centralised repository of lab results enables tracking patients’ profiles and helps reduce unnecessary repetitive examinations.

### Bulgaria: Integrated cardiovascular prevention

Alignment of its cardiovascular prevention strategy with European frameworks introduced targeted high-risk programmes (ProAction BG and REVEALED) and established lipid centres with early FH screening.

## Cross-Cutting Challenges Identified

Across both meetings, several systemic gaps were repeatedly emphasised:

- Low public and provider awareness of cholesterol risks.
- Limited FH and Lp(a) testing, including resistance to infant FH screening.
- Inadequate data systems, hampering policy advocacy and investment.
- Fragmented care pathways, especially in primary care.
- Underuse of statins and combination therapies despite their effectiveness.
- Lack of reimbursement or inconsistent coverage of advanced therapies.
- Political and cultural barriers, including scepticism and misinformation.

## Recommendations for Strengthening Lipid Management across Europe

Drawing on the roundtable findings, the following recommendations emerge for national and regional action to strengthen lipid management in Europe.

### 1. Strengthen national policies and funding mechanisms

- Establish or update national lipid management strategies.
- Integrate FH and Lp(a) into national CVD screening frameworks.
- Prioritise reimbursement pathways for high-risk groups.
- Leverage cost-effectiveness evidence in advocacy efforts.

### 2. Improve data systems and monitoring

- Collect and report national data on screening, treatment, and control rates.
- Develop FH and Lp(a) registries.
- Use quality metrics to benchmark regional performance.
- Support real-world implementation studies to guide policy adaptation.

### 3. Enhance primary care capacity

- Provide training on statin intolerance, FH detection, risk assessment, and Lp(a).
- Introduce simplified referral tools and electronic pathways.
- Integrate lipid management into primary care quality frameworks.

### 4. Promote public and patient education

- Counter misinformation with evidence-based campaigns.
- Use behaviourally informed communication strategies.
- Engage patients in designing and delivering awareness programmes.

### 5. Expand access to essential and advanced therapies

- Ensure inclusion of combination therapies in essential medicines lists.
- Advocate for reimbursement for advanced therapies for high-risk patients.
- Align national decisions with European and international guidelines.

### 6. Foster cross-country collaboration

- Support shared learning and coordinated action across Europe.
- Encourage EU-level patient advisory boards to inform policy.
- Use the Spanish and Polish models as potential templates for scale-up.

## Conclusion

Improving lipid management across Europe requires strengthening health systems, addressing cultural and informational barriers, expanding access to therapies, and elevating the detection and management of inherited lipid disorders. The WHF Regional Europe Roundtable Series demonstrates that despite diverse national contexts, many challenges are shared, and many solutions are transferable. By adopting evidence-based strategies, investing in data and infrastructure, and fostering collaboration across countries, Europe can make substantial progress toward reducing cardiovascular risk and achieving lipid targets for all high-risk populations.
